# The Deficits of Individual Morphological Covariance Network Architecture in Schizophrenia Patients With and Without Violence

**DOI:** 10.3389/fpsyt.2021.777447

**Published:** 2021-11-15

**Authors:** Danlin Shen, Qing Li, Jianmei Liu, Yi Liao, Yuanyuan Li, Qiyong Gong, Xiaoqi Huang, Tao Li, Jing Li, Changjian Qiu, Junmei Hu

**Affiliations:** ^1^Mental Health Center, West China Hospital, Sichuan University, Chengdu, China; ^2^Qingyang Yalun Clinic, Chengdu, China; ^3^Department of Radiology, West China Second University Hospital, Sichuan University, Chengdu, China; ^4^Huaxi MR Research Center (HMRRC), West China Hospital, Sichuan University, Chengdu, China; ^5^Affiliated Mental Health Center, School of Medicine, Zhejiang University, Hangzhou, China; ^6^School of Basic Science and Forensic Medicine, Sichuan University, Chengdu, China

**Keywords:** individual morphological covariance network, graph theory, schizophrenia, violent, gray matter volume

## Abstract

**Background:** Schizophrenia is associated with a significant increase in the risk of violence, which constitutes a public health concern and contributes to stigma associated with mental illness. Although previous studies revealed structural and functional abnormalities in individuals with violent schizophrenia (VSZ), the neural basis of psychotic violence remains controversial.

**Methods:** In this study, high-resolution structural magnetic resonance imaging (MRI) data were acquired from 18 individuals with VSZ, 23 individuals with non-VSZ (NSZ), and 22 age- and sex-matched healthy controls (HCs). Whole-brain voxel-based morphology and individual morphological covariance networks were analysed to reveal differences in gray matter volume (GMV) and individual morphological covariance network topology. Relationships among abnormal GMV, network topology, and clinical assessments were examined using correlation analyses.

**Results:** GMV in the hypothalamus gradually decreased from HCs and NSZ to VSZ and showed significant differences between all pairs of groups. Graph theory analyses revealed that morphological covariance networks of HCs, NSZ, and VSZ exhibited small worldness. Significant differences in network topology measures, including global efficiency, shortest path length, and nodal degree, were found. Furthermore, changes in GMV and network topology were closely related to clinical performance in the NSZ and VSZ groups.

**Conclusions:** These findings revealed the important role of local structural abnormalities of the hypothalamus and global network topological impairments in the neuropathology of NSZ and VSZ, providing new insight into the neural basis of and markers for VSZ and NSZ to facilitate future accurate clinical diagnosis and targeted treatment.

## Introduction

Schizophrenia (SZ) is a serious mental disorder affecting 1% of the world's population in terms of thinking, feeling, and behaviors that cause abnormal perceptions of reality (1). The link between SZ and violent offending has long been the subject of research with a significant impact on mental health policy. Patients with SZ have an elevated risk for aggression and violent behavior, which leads to fear and contributes to the major stigma of this disease (2). Although previous studies have reported that environmental factors, such as low socio-economic status and childhood trauma, may lead to violence in SZ (3–5), increasing evidence indicates that neurobiological factors may also play a key role in the increased risk of violence in individuals with SZ (6, 7). The origins of violent behavior in people with SZ are not yet sufficiently understood (8). Moreover, the management of aggression in SZ patients is a challenging clinical dilemma given that violence or aggressive behavior is heterogeneous in origin (8–10). Therefore, delineating the underlying neurobiological basis of violence in SZ may facilitate its management and effective therapy.

Non-invasive magnetic resonance imaging (MRI) provides the opportunity to study brain structure and function *in vivo*. Widely used structural MRI (sMRI), diffusion MRI, and functional MRI enable investigations of brain morphology, white matter (WM) microstructure, and functional activities, respectively (11–17). In recent decades, mounting studies have demonstrated that brain function is not only fulfilled by a single area but also involves interactions across multiple distributed systems to form a complex brain network (18–22). Traditionally, brain networks were mapped using diffusion MRI for axonal connections or functional MRI for functional connectivities (23–28). Recently, using sMRI to map whole-brain morphological connectivity patterns by characterizing interregional morphological similarities was proposed due to its advantages of easy access, high signal-to-noise ratio, and robustness to artifacts (29–31). Unlike four-dimensional functional MRI, sMRI only contains three-dimensional location information. Early studies thus constructed the morphological covariance network at the population level by taking each individual subject as a time point to model time series of functional MRI (30, 32). A group of subjects can only obtain one connectivity matrix to reflect the group-level morphological covariance, ignoring individual variability. Recently, Wang et al. (33) developed an individual morphological covariance network method used for brain disease research (34, 35). Thus, individual morphological covariance networks with graph theory analysis may provide new insight into brain network organization patterns and improve the understanding of the neurobiological underpinnings of violent SZ (VSZ) patients.

In the current study, we aimed to explore structural and topological differences in gray matter volume (GMV) and individual morphological covariance networks among healthy controls (HCs) and individuals with non-VSZ (NSZ) and VSZ. In addition, we evaluated the associations of changes in GMV and network topology with clinical variables.

## Materials and Methods

### Participants

This study was approved by the Ethics Committee of West China Hospital, Sichuan University. A total of 18 VSZ, 23 NSZ, and 22 HCs participated in the present study. All the subjects were male and were matched based on age and education level among the three groups. All participants were right-handed, and written informed consent was provided and obtained. All subjects were recruited from the forensic psychiatry department of Preclinical Science and Forensic Medicine College of Sichuan University, Chengdu, Sichuan. Psychiatric diagnoses were determined by two experienced psychiatrists using the Structured Clinical Interview for *Diagnostic and Statistical Manual of Mental Disorders*, Fourth Edition (DSM-IV) (SCID-I/P), Chinese version. The inclusion criteria for VSZ were murder, attempted murder, and severe physical assault toward other people (including sexual assaults) based on the MacArthur criteria (36). These individuals committed serious violence with at least one fatal or near-fatal act of violence against their victims and were referred to forensic psychiatric examination for legal competence before court decisions. All participants were diagnosed with SZ before receiving any medical treatment. The exclusion criteria were (1) age <18 years or over 65 years; (2) other psychiatric co-morbidities; (3) any history of cardiovascular diseases, major physical illness, or neurological disorder; and (4) substance abuse or dependence. Brain MRI performed under the supervision of an experienced neuroradiologist showed no gross abnormalities.

### Clinical Assessments and Criminal Information

Psychopathology was assessed using the Chinese version of the Positive and Negative Syndrome Scale (PANSS) (37), which provides a total score and positive, negative, and general symptoms, and supplement scores. The Chinese version of the PANSS consists of the original PANSS plus three supplementary excitability items, including anger, difficulty in delay gratification, and affective liability, to measure the excitement dimension. The supplement scores were not added to the PANSS total score. The assessments were conducted by clinical psychiatrists who were professionally trained to conduct the PANSS interview and employ rating methods. Individual incidents of aggression were recorded using self-reporting criminology-characterized tables and modified overt aggression scale (MOAS) (38). The tables characterized by self-reported criminology include types of cases, attack targets, preparation of crime, criminal motivation, and self-protection. All of the information was collected based on criminal case files.

### Structural MRI Data Acquisition

sMRI data were acquired using a 3-Tesla Siemens MRI system with an eight-channel phase-array head coil. Head motion was controlled using foam pads. Prior to scanning, participants were instructed to lie still with their eyes closed and not to fall asleep. High-resolution T1-weighted data were acquired using the following scan parameters: repetition time (TR) = 1,900 ms, echo time (TE) = 2.28 ms, flip angle = 9°, 176 sagittal slices with slice thickness = 1.0 mm, field of view = 240 × 240 mm^2^, and data matrix = 256 × 256.

### Voxel-Based Morphometry Analyses

The sMRI images were processed using the CAT12 toolbox in SPM12 software (http://dbm.neuro.uni-jena.de/wordpress/vbm/download/). Voxel-based morphometry (VBM) analysis included the following steps. MRI images were first assessed to exclude artifacts or gross anatomical abnormalities and were reoriented to the anterior commissure. Then, the structural images were segmented into GM, WM, and cerebrospinal fluid (CSF). Next, GM images were normalized to the Montreal Neurological Institute (MNI) space using the Diffeomorphic Anatomical Registration using Exponentiated Lie algebra (DARTEL) normalization approach and were modulated to account for volume changes. Finally, the GM images were smoothed using a Gaussian kernel of 8-mm full-width at half maximum (FWHM) (32, 39), and whole-brain voxelwise one-way ANOVA with PANSS and disease duration as covariates was performed to identify differences in GMV among HCs, NSZ, and VSZ. The significance level was set as *p* < 0.05 using false discovery rate (FDR) correction and a minimum cluster size of >30. After identification of GMV differences, the mean GMV in brain areas with altered GMV in the HC, NSZ, and VSZ groups was calculated. *Post-hoc* two-sample *t*-tests were further used to identify between-group differences, and the significance was set at *p* < 0.05 with Bonferroni correction.

### Individual Morphological Covariance Network Analysis

#### Defining Network Nodes

To explore brain network topology changes across different groups, individual morphological covariance networks were studied with GM images in the template space for each subject. The brain network includes network nodes and network edges. In this study, network nodes were defined with automated anatomical labeling (AAL) atlases (40). Each cortical and subcortical subregion served as a node in the morphological covariance network.

#### Defining Network Edges

After the nodes of the morphological network were defined, the edge was defined as the interregional similarity in the distribution of the regional GMV. The edge of the individual morphological covariance network was calculated as follows: kernel density estimation (KDE) was first used to estimate the probability density function of the extracted GMV values of each subregion in the AAL atlas, and the variation in the Kullback–Leibler (KL) divergence (KLD) was calculated to define the similarity of GM values between each pair of subregions. The similarities were taken as the edges of the morphological covariance network (33). Given that the AAL atlas segments the cortex and subcortex into 90 subregions, a 90 × 90 matrix was obtained for each subject. Finally, a binary network was generated for each subject for further analyses.

### Graph Theory-Based Network Analyses

To explore network topology parameter differences among HCs, NSZ, and VSZ, graph theory-based network analyses were performed with sparsity values from 0.05 to 0.39 using steps of 0.02. First, small worldness was assessed for each morphological covariance network. If each morphological covariance network met the small-world property (normalized clustering coefficient >> 1, normalized characteristic path length ≈ 1, and small worldness > 1), the global and nodal topological parameters, including clustering coefficient (Cp), global efficiency (Eg), local efficiency (Eloc), shortest path length (Lp), assortativity, modularity, nodal degree, and nodal betweenness, were calculated. One-way ANOVA with PANSS and disease duration as covariates was first used to identify differences in network parameters among HCs, NSZ, and VSZ; and the significance level was set at *p* < 0.05. *Post-hoc* two-sample *t*-tests were further used to determine between-group differences corrected with the Bonferroni method with *p* < 0.05.

### Correlation Analyses

To explore whether GMV and network topology abnormalities were associated with illness duration, PANSS, and aggression, correlation analyses were conducted in NSZ and VSZ patients. The significance level was set at *p* < 0.05 corrected using the FDR method.

## Results

### Demographic and Clinical Information

No significant differences in age (*p* = 0.53) or education (*p* = 0.38) were noted among the HC, NSZ, and VSZ groups as shown in Table 1. Patients with VSZ had longer disease duration (*p* = 0.0063), higher PANSS (*p* < 0.001), and higher aggression scores (*p* < 0.001) than NSZ patients (Table 1).

**Table 1:** Subject demographics.

	**HC (*n* = 22)**	**NSZ (*n* = 23)**	**VSZ (*n* = 18)**	***F*/*t* values**	***p*-values**
Age (years)	32.36 (4.93)	31.22 (6.54)	33.61 (8.62)	0.64	0.53
Sex (M/F)	22/0	23/0	18/0	NA	NA
Education (years)	12.48 (2.61)	12.78 (2.98)	11.56 (2.97)	0.98	0.38
Duration (months)	NA	16.1 (28.88)	59.89 (65.25)	2.89	0.0063^*^
PANSS	NA	86.3 (16.49)	112 (7.4)	6.13	<0.001^*^
MOAS	NA	14.96 (3.99)	29 (3.33)	12	<0.001^*^

*HCs, healthy controls; NSZ, nonviolent schizophrenia; VSZ, violent schizophrenia; M, male; F, female; PANSS, Positive and Negative Syndrome Scale; MOAS, modified overt aggression scale*.

* *Significant differences*.

### Abnormal Gray Matter Volume

**Figure 1:**
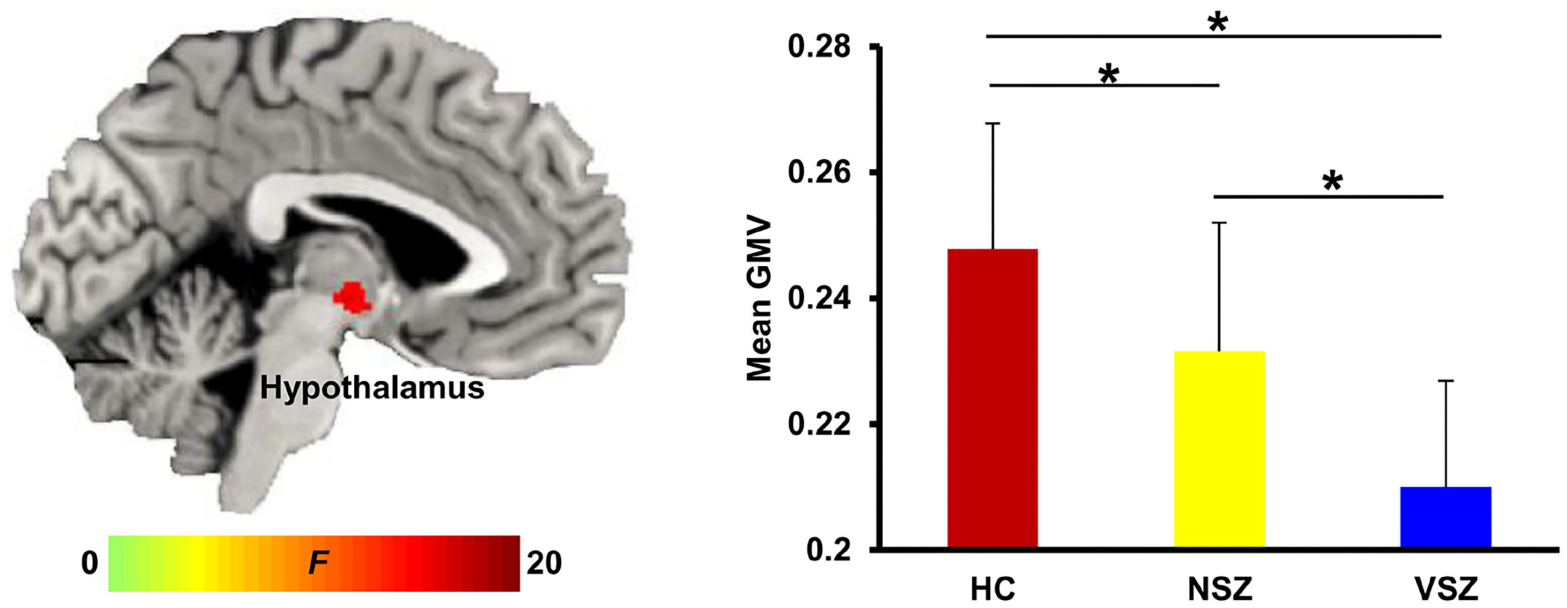
Abnormal gray matter (GM) volume in the hypothalamus was found among healthy controls (HCs), nonviolent schizophrenia (NSZ) patients, and violent schizophrenia (VSZ) patients. A gradient decrease in GM volume from HCs to NSZ and VSZ was observed. *Significant difference with *p* < 0.05.

Abnormal GMV in the right hypothalamus (peak coordinate, x = 3, y = −11, z = −6) was noted among the HC, NSZ, and VSZ groups. *Post-hoc* two-sample *t*-tests found significantly lower GMVs in SZ patients compared with HCs and significantly lower GMVs in VSZ patients compared with NSZ patients (Figure 1).

### Abnormal Network Topology

**Figure 2:**
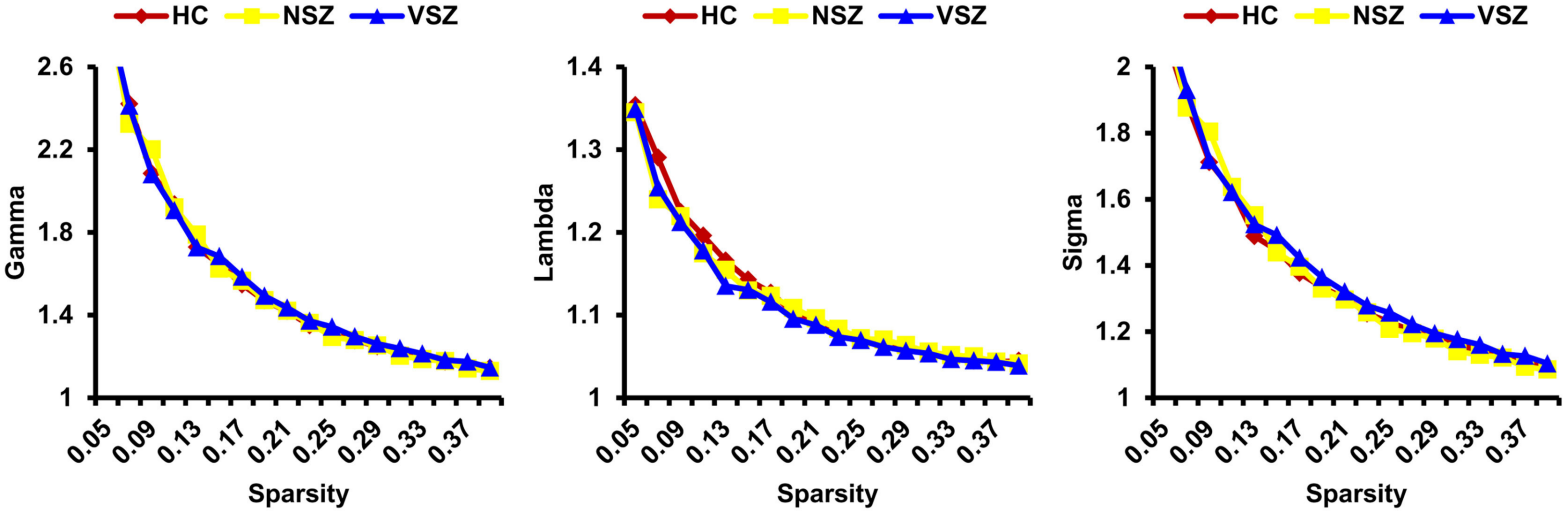
All the HC, NSZ, and VSZ groups showed small-worldness properties of the individual morphological covariance network at sparsity values ranging from 0.05 to 0.39. HC, healthy control; NSZ, nonviolent schizophrenia; VSZ, violent schizophrenia.

**Figure 3:**
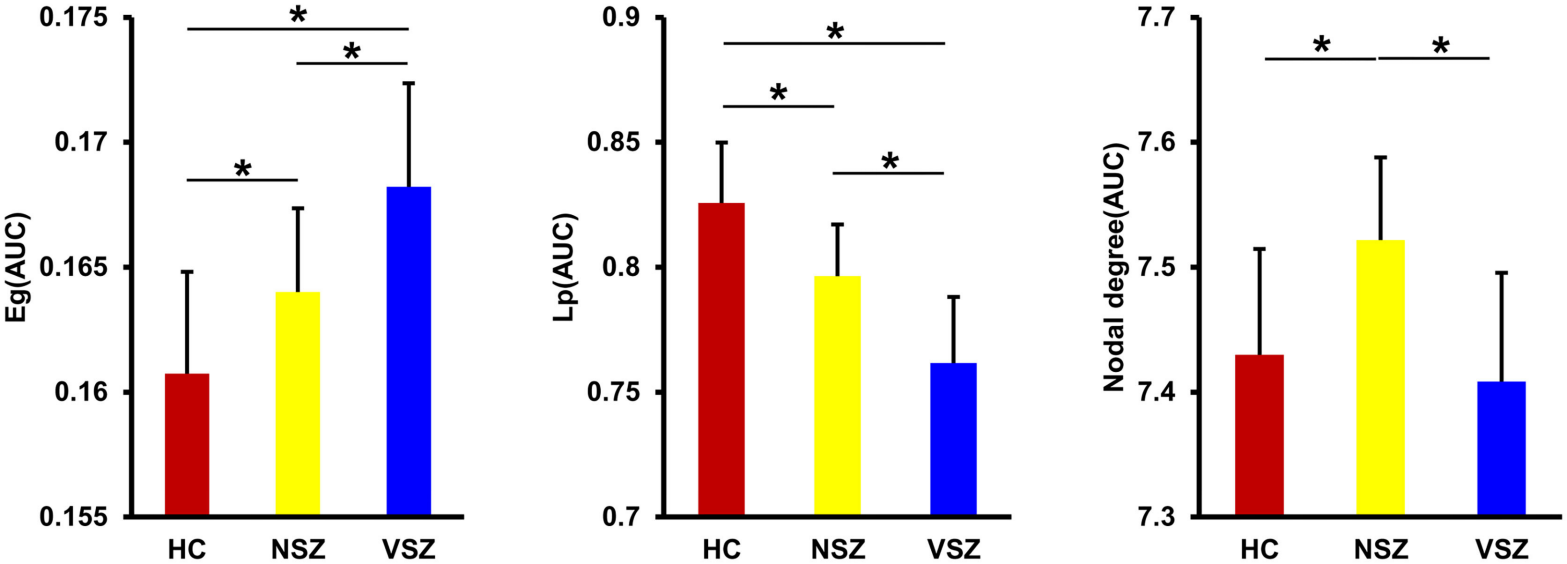
Significant differences in global efficiency (Eg), shortest path length (Lp), and nodal degree were found among the HC, NSZ, and VSZ groups. A gradient increase in Eg and a gradient decrease in Lp from HCs to NSZ and VSZ were found. NSZ had a significantly higher nodal degree than both HCs and VSZ. * Significant difference with *p* < 0.05. HC, healthy control; NSZ, nonviolent schizophrenia; VSZ, violent schizophrenia.

All the morphological covariance networks of HCs, NSZ, and VSZ showed small-worldness properties at sparsity values ranging from 0.05 to 0.39 (Figure 2). Abnormal network topological parameters, including Eg, Lp, and nodal degree, were found among the HC, NSZ, and VSZ groups (Figure 3). Both NSZ and VSZ patients showed significantly higher Eg than HCs; and VSZ individuals had significantly higher Eg than NSZ individuals. For Lp, both NSZ and VSZ patients exhibited significantly lower Lp than HCs, and VSZ exhibited significantly lower Lp than NSZ. The mean nodal degree in NSZ individuals was significantly greater than that observed in HCs and VSZ, but no significant difference was noted between HCs and VSZ. For other network topological parameters, including small worldness (Gamma, Lambda, and Sigma), Eloc, assortativity, modularity, Cp, and nodal betweenness, no significant differences were noted among HCs, NSZ, and VSZ (Supplementary Figure 1).

### Correlation Analysis Results

**Figure 4:**
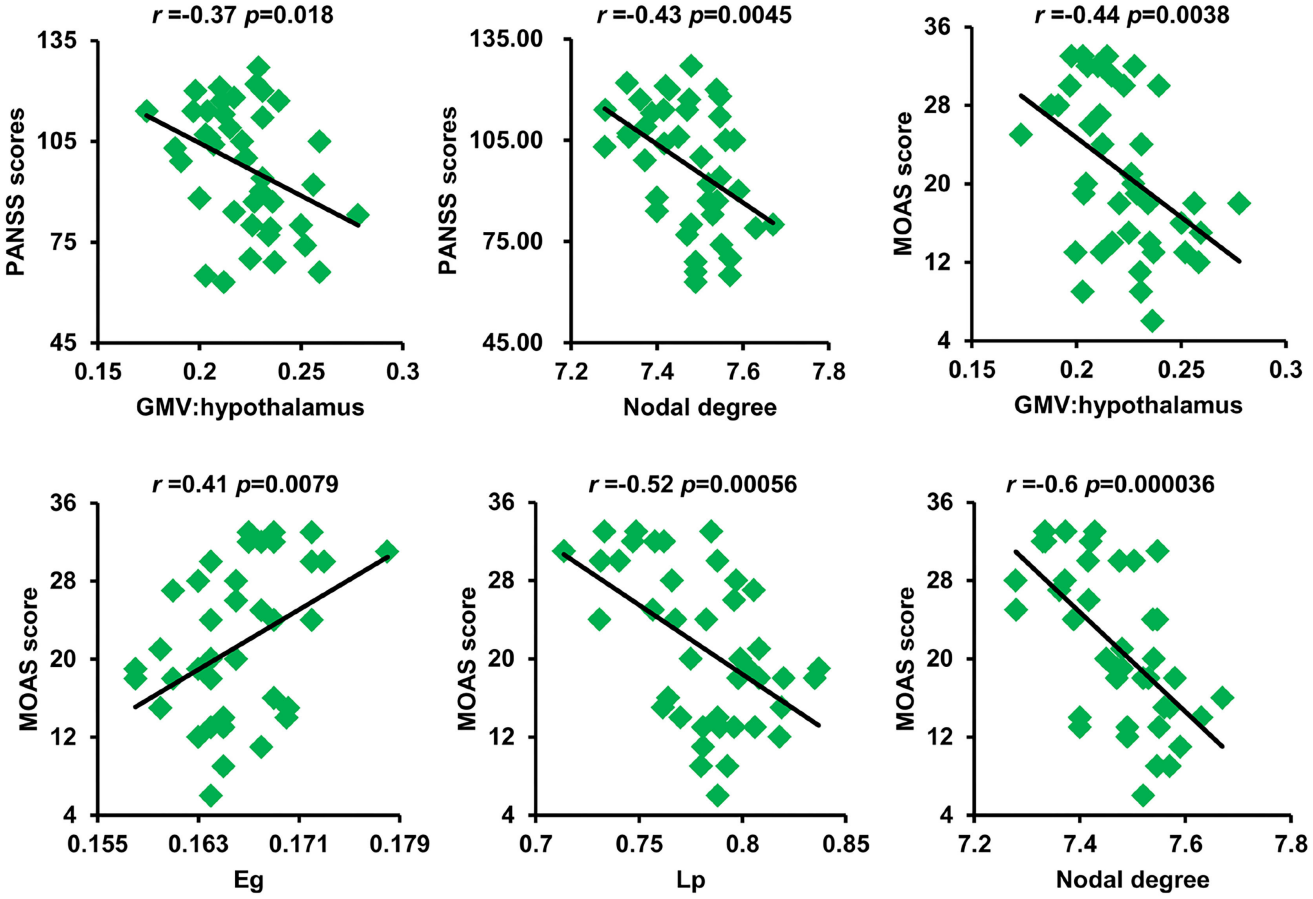
Significant correlations between GM volume of the hypothalamus and nodal degree and PANSS scores as well as between GM volume of the hypothalamus and Eg, Lp, nodal degree, and MOAS scores were found. GM, gray matter; PANSS, Positive and Negative Syndrome Scale; Eg, global efficiency; Lp, shortest path length; MOAS, modified overt aggression scale.

As shown in Figure 4, after correction for multiple comparisons, GMV of the hypothalamus and nodal degree showed significantly negative correlations with PANSS scores. The GMV of the hypothalamus, Lp, and nodal degree showed significantly negative correlations with aggression scores, whereas Eg exhibited a significantly positive correlation with aggression scores.

## Discussion

In the current study, we revealed alterations in GMV and disrupted network topology in SZ patients with and without violence using voxel-based morphology and novel individual morphological covariance network analyses. Significantly decreased GMV in the hypothalamus and significantly disrupted global efficiency, short path length, and nodal degree were found in the NSZ and VSZ groups. Moreover, these changed GMVs and network topologies were significantly associated with clinical characteristics. These findings highlighted the important role of the hypothalamus in SZ patients with and without violence and deepened our understanding of the neuropathology of SZ and violence in SZ from a network perspective.

The hypothalamus is the control center for many autonomic functions of the peripheral nervous system and plays a vital role in maintaining homeostasis (41–43). As a part of the limbic system, the hypothalamus also influences various emotional responses (44). Many recent studies have demonstrated that the hypothalamus is important for circadian control aggression in both humans and animals (45–47). Decreased hypothalamus volume has been reported in patients with SZ (48–50). The decreased GMV in the hypothalamus supported our finding of decreased GMV in NSZ patients compared with HCs. However, few studies have reported abnormal GMV in the hypothalamus in SZ patients with aggression. Only one study by Schiffer et al. (51) found increased GMV in the hypothalamus in patients with SZ with conduct disorder, which is associated with violent behavior. In our study, we found decreased GMV in individuals with VSZ. The difference may result from different subtypes of VSZ, suggesting that different subtypes of VSZ may have distinct neural circuits. Moreover, we found that the GMV gradually decreased from HCs to NSZ and VSZ and was significantly correlated with PANSS and aggression scores in patients. These findings indicate that abnormal GMV of the hypothalamus may be an intrinsic biomarker to distinguish individuals with SZ from healthy individuals and to differentiate individuals with VSZ and NSZ.

The human brain is conceptualized as a complex network structured to optimize the interplay between segregation and integration of functionally specialized subsystems (19, 52). Many previous studies have utilized diffusion MRI or functional MRI to map anatomical or functional brain networks to explore network topological abnormalities in SZ (53–57). By measuring across-subject covariance in morphological measures, such as cortical thickness (29), gyrification (58), and GMVs (31, 59), structural network topological attributes were also studied in SZ. Although the GMV covariance network has been analyzed in SZ, all previous studies use population-level data to construct only one single connectivity network across all subjects, which cannot account for individual network topology. To the best of our knowledge, this is the first study to map the individual morphological covariance network to investigate abnormal network topology in VSZ and NSZ. We found gradually increased global efficiency and gradually decreased shortest path length from HCs to NSZ and VSZ. We also found an increased mean nodal degree in NSZ individuals compared with both HC and VSZ individuals. The findings in our study were supported by previous complex brain network analyses in SZ (54, 56, 60). All the evidence suggested higher information processing efficiency in NSZ and VSZ individuals compared with HCs. Our results together with previous findings may support the “hyperconnectivity” hypothesis of SZ (61–63). In addition, abnormal global efficiency, shortest path length, and nodal degree were significantly correlated with PANSS and aggression scores. Thus, global efficiency and shortest path length may serve as biomarkers to distinguish individuals with VSZ and NSZ, whereas nodal degree may be a specific neurobiomarker for NSZ.

The current study also has several limitations. First, in our study, the sample size was limited, and all the subjects were male. These results need to be interpreted with caution; thus, the findings in our study also require further validation. Second, longitudinal studies are warranted to better reveal the neuropathology of NSZ and VSZ using multimodal MRI data and to extend the findings in further studies.

In conclusion, our study found a gradual decrease in GMV in the hypothalamus and disrupted global topological properties, including global efficiency, shortest path length, and nodal degree, in individuals with VSZ and NSZ. In addition, we found that abnormal GMV and network topological properties were significant clinical measures. These findings highlight the important roles of the hypothalamus in individuals with VSZ and NSZ and provide neural biomarkers to distinguish SZ from healthy subjects and to differentiate subtypes of SZ.

## Data Availability Statement

The raw data supporting the conclusions of this article will be made available by the authors, without undue reservation.

## Ethics Statement

The studies involving human participants were reviewed and approved by Ethics Committee of West China Hospital, Sichuan University. The patients/participants provided their written informed consent to participate in this study.

## Author Contributions

All authors listed have made a substantial, direct and intellectual contribution to the work, and approved it for publication.

## Conflict of Interest

The authors declare that the research was conducted in the absence of any commercial or financial relationships that could be construed as a potential conflict of interest.

## Publisher's Note

All claims expressed in this article are solely those of the authors and do not necessarily represent those of their affiliated organizations, or those of the publisher, the editors and the reviewers. Any product that may be evaluated in this article, or claim that may be made by its manufacturer, is not guaranteed or endorsed by the publisher.
