# Supplementary material for: The Deficits of Individual Morphological Covariance Network Architecture in Schizophrenia Patients With and Without Violence
Source: Front Psychiatry. 2021 Nov 15;12:777447. doi: 10.3389/fpsyt.2021.777447 (PMC8634443; doi:10.3389/fpsyt.2021.777447)

**Figure S1.** Statistical analyses of other network topological parameters including small worldness properties (Gamma, Lambda, and Sigma), local efficiency (Eloc), assortativity, clustering efficiency (Cp), modularity, and nodal betweenness were performed among healthy controls (HC), non-violent (NSZ), and violent schizophrenia (VSZ). No significant differences were found.


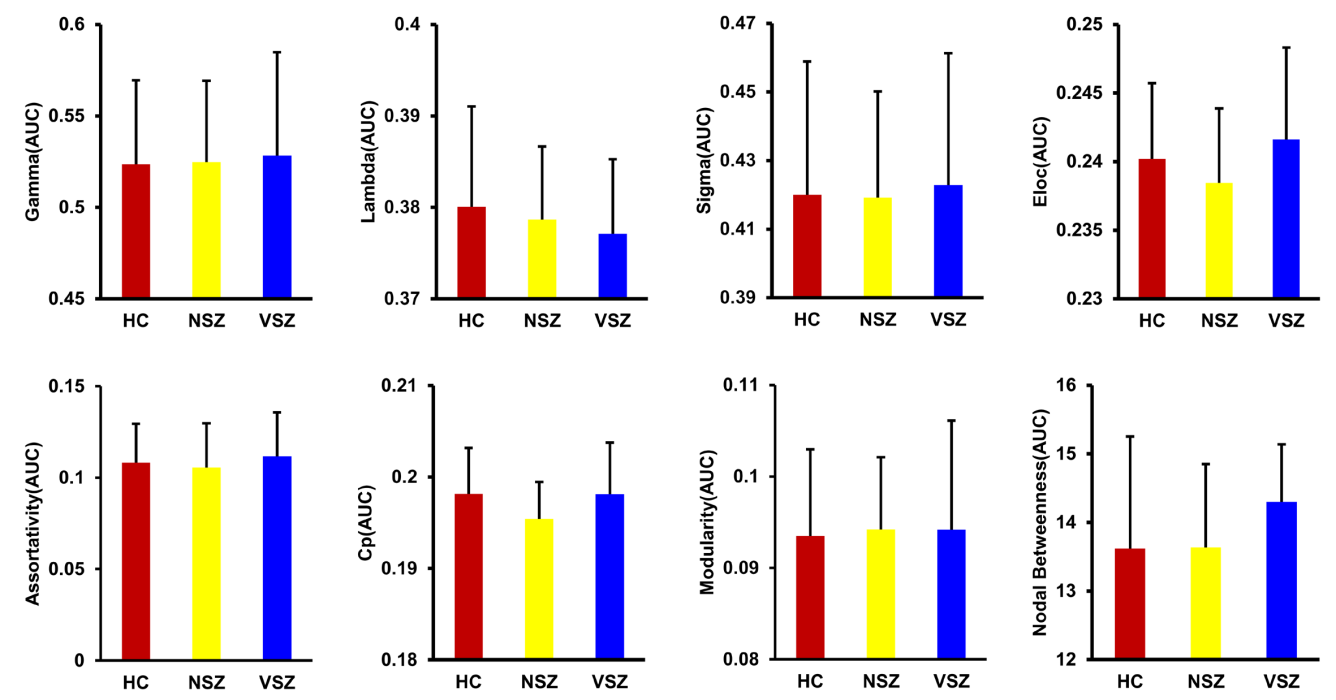

Supplement: Supplementary file 1 [file Data_Sheet_1.docx]
